# Large chest keloids treatment with expanded parasternal intercostal perforator flap

**DOI:** 10.1186/s12893-021-01116-3

**Published:** 2021-03-20

**Authors:** Hao Liu, Fuqiang Sui, Shu Liu, Kexin Song, Yan Hao, Youbin Wang

**Affiliations:** 1 Department of Plastic Surgery, Peking Union Medical College Hospital, Beijing, China; 2 Department of Burn and Plastic Surgery, 969 Hospital Associated With Logistic Support Forces of the Chinese People’s Liberation Army, Neimenggu, China; 3 Department of General Surgery, Civil Aviation General Hospital, Beijing, China; 4 Chiese Academy of Medical Sciences, Peking Union Medical College, Beijing, China

**Keywords:** Keloid, Expanded parasternal intercostal perforator flap (EPIPF), Surgical treatment, Radiotherapy

## Abstract

**Background:**

Chest keloids often converged into a large lesion on the chest in some patients. Such keloids often lead to obstacle to excision and reconstruction. We describe a surgical method for large chest keloids with expanded parasternal intercostal perforator flap (EPIPF).

**Methods:**

Fifteen patients with chest keloid were treated with EPIPF in our department between August 2017 and Dec 2019. The surgical treatment was divided into two different phases. In the first phase, we implanted skin expanders into the layer under the deep fascia beside the keloids. The expander was expanded every week for about 3–4 months. In the second phase, the expander was removed, the keloid tissue was removed and an expanded perforator flap was then designed to cover the wound. Patients were followed-up after surgery. Complications after surgery were analyzed. Recurrence and the patients, satisfactory rate was recorded.

**Results:**

Of the 15 patients, one patient complicated with undesirable small area wound healing. 11 were cured without scar hypertrophy or recurrence and four were partially cured with a small portion of scar hypertrophy. Eleven patients thought that the esthetic result was good (73.7%), and 4 patients thought the result was acceptable (26.7%). None patient was dissatisfied.

**Conclusion:**

EPIPF are effective surgical method for managing large chest keloids. It can offer enough skin flap coverage for keloid wound resurfacing with stable blood supply to assure satisfactory results.

**Level of evidence:**

Level IV, case series.

## Background

Keloids represent a kind of superficial benign tumor that grows aggressively for response to dermal injury, which was characterized by overproduction of collagen and local fibroblast proliferation [1]. Patients suffered from keloids often complain of unwanted symptoms such as pruritus, pain, skin infection and aesthetic problems, these symptoms can be especially severe during the growth phase, which presents a significant burden for the patients [2].

Nowadays, there are many therapeutic options for clinicians, including silicone-based therapy, intralesional steroids, cryosurgery, 5-fluorouracil, interferon, lasers, verapamil, radiotherapy and surgery [3–5]. The clinician should choose the most appropriate method based on the symptoms of the patient.

Chest keloid is common in clinical practice. The shape and size are often different among the patients. Some small keloids are round and oval. These can be removed directly in one surgery. Some are large and irregular. Such keloids are often difficult for surgical treatment. We have reported using radiotherapy before skin grafting or transfer parasternal intercostal perforator flap even microsurgery with superficial circumflex iliac artery perforator (SCIP) skin flap to reconstruct the wound after resecting the lesion [6, 7, 10]. However, for giant irregular and “crab claw” like keloids, all these methods are not the most appropriate. Lack of skin tissue to cover the wound after resecting the keloid lesion remains to be one of the biggest problems in keloid surgical treatment. In the study, we reported the outcome of 15 patients who has giant keloid of chest and been treated with pre-expanded parasternal intercostal perforator flap after keloid resection in our hospital.

## Methods

### Materials

We retrospectively analyzed the data of patients with chest keloid and accepting surgical treatment in Department of Plastic Surgery, Peking Union Medical College Hospital, Beijing, China between August 2017 and Dec 2019. We retrieved the demographic data of patients about keloid sizes, flap sizes, complications and recurrences from the medical records.

The mean age of all the patients was about 40 years old (range 22–63 years old). 12 cases of them were caused by local repeated infection, 3 cases were caused by no apparent reason. The keloid size was about from 8 cm × 6 cm to 21 cm × 15 cm.

In this study, all the protocols were approved by Peking Union Medical College Hospital ethics review committee. All the patients were anonymized in this study.

### Surgical methods

All the patients mentioned in this study provided written informed consent to the surgery. All the surgical procedures were performed by the same surgical team with more than 15 years of experience in plastic surgery.

**Fig. 1:**
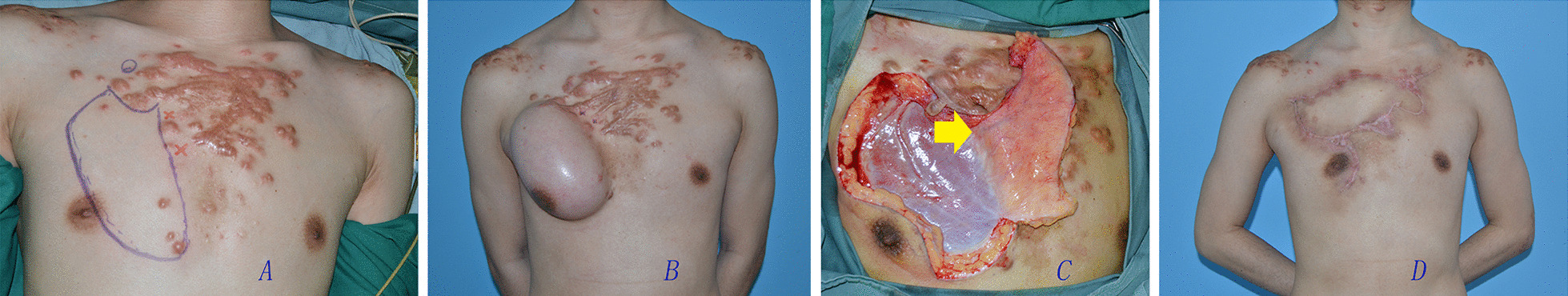
Chest keloid treated with EPIPF

The surgical treatment was divided into two different phases. In the first phase, we implanted skin expanders (Size: from 100 to 300 ml, Rectangle) to the depth beneath the layer of deep fascia beside keloids. Ultrasonic Doppler was used to determine the site and the course of the intercostal perforator vessel to assure the vessel was in the scope of the expanded skin. (Fig. 1a) In patient with very large and irregular keloid, two or more expanders were implanted. After the expander was properly implanted, sterile saline solution was injected into the expanders once a week, ten percent of the expander volume each time in each expander. After 3 to 4 months expanding, the skin can be expanded to appropriate size to cover the wound after keloid resection. (Fig. 1b) Then we performed phase two surgery. In this phase, incisions were made in the skin surrounding keloid and keloid tissue was removed in the depth of the layer between deep fascia and superficial fascia. The intercostal perforating vessels were preserved by cautious surgical procedures. Then the expander was removed. An EPIPF was then designed according to wound after keloid resection. The flap was then fully elevated while preserving the perforator (Fig. 1c) and rotated smoothly to fit with the recipient site. The donor site and the wound after keloid resection was then closed. Continuous suction drains were placed under the flap and the donor site.

### Postoperative therapy

Two sessions of radiotherapy were performed after phase two surgery, the first session was performed 24 h after surgery, the second session was on the eighth postoperative day, 9 Gy in each session. The suction drains were removed within 2–4 days after surgery. 14 days after surgery in phase 2, all the dressings and sutures were removed. Continuous pressure therapy was applied for 6 to 12 months. All the patients were followed up by scheduled visits every 3–6 months after surgery.

## Results

**Fig. 2:**
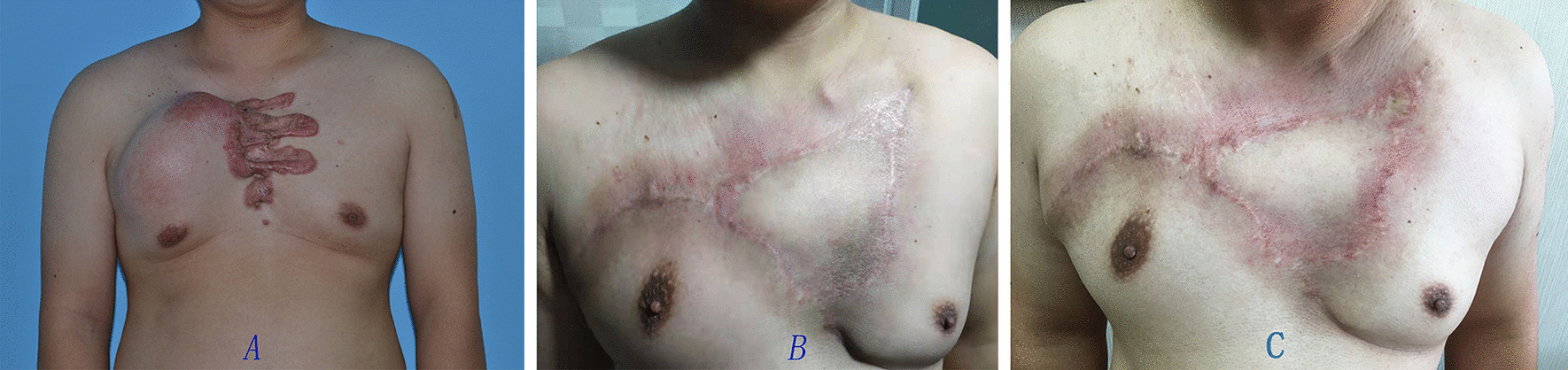
Chest keloid treated with EPIPF

**Fig. 3:**
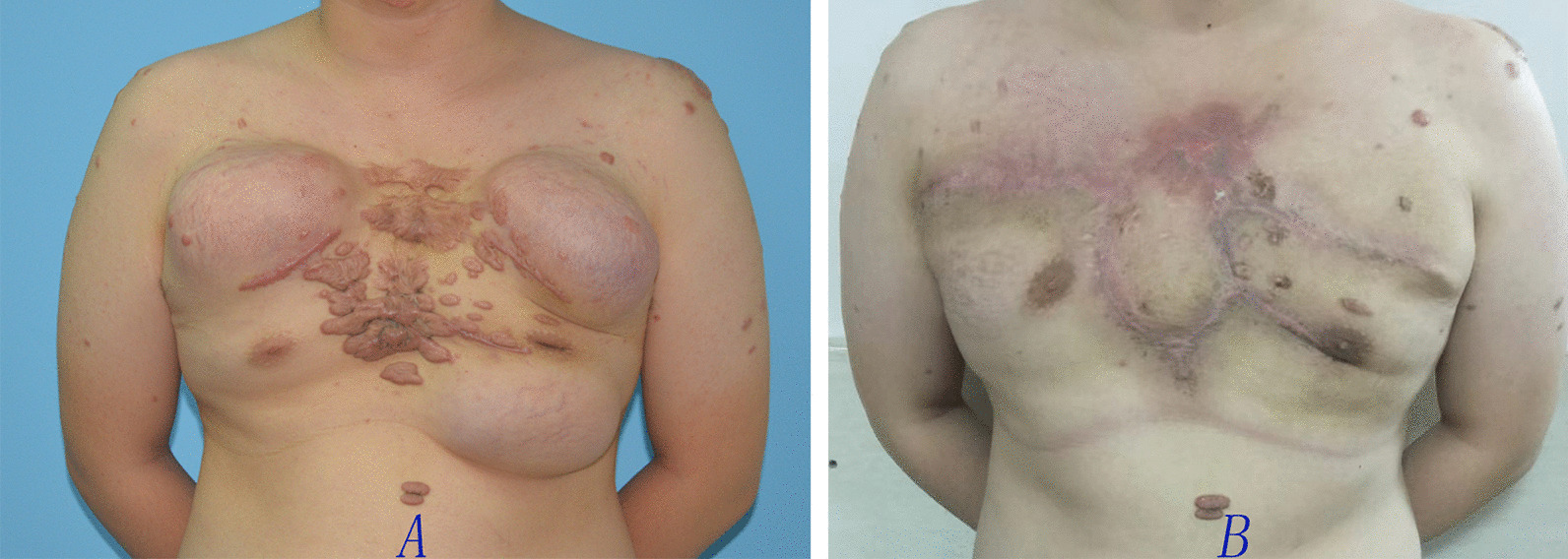
Chest keloid treated with EPIPF

**Fig. 4:**
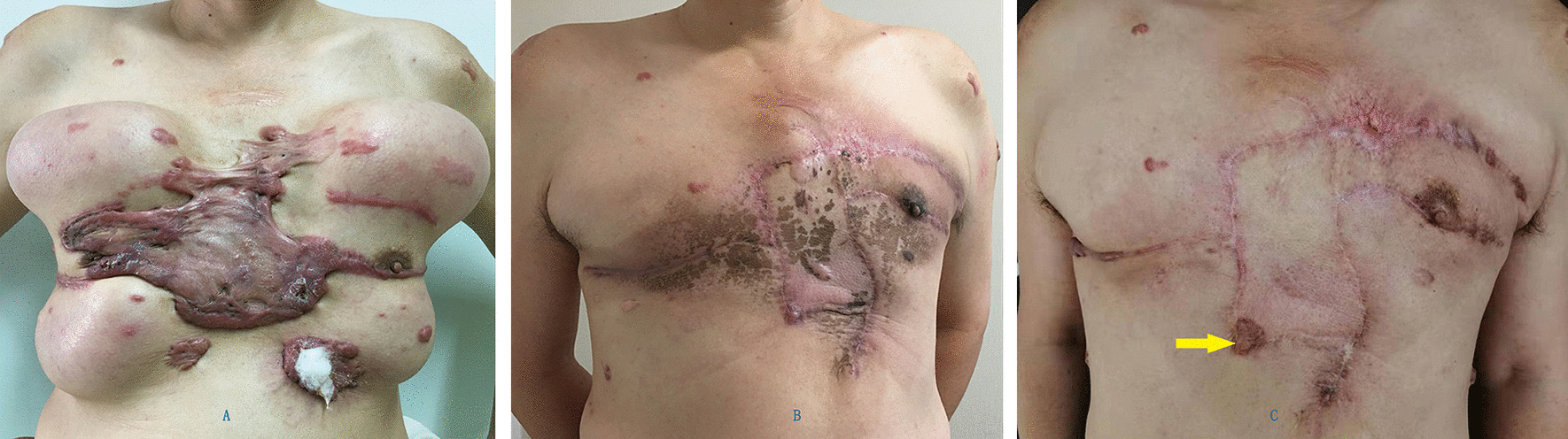
Chest keloid treated with EPIPF

**Fig. 5:**
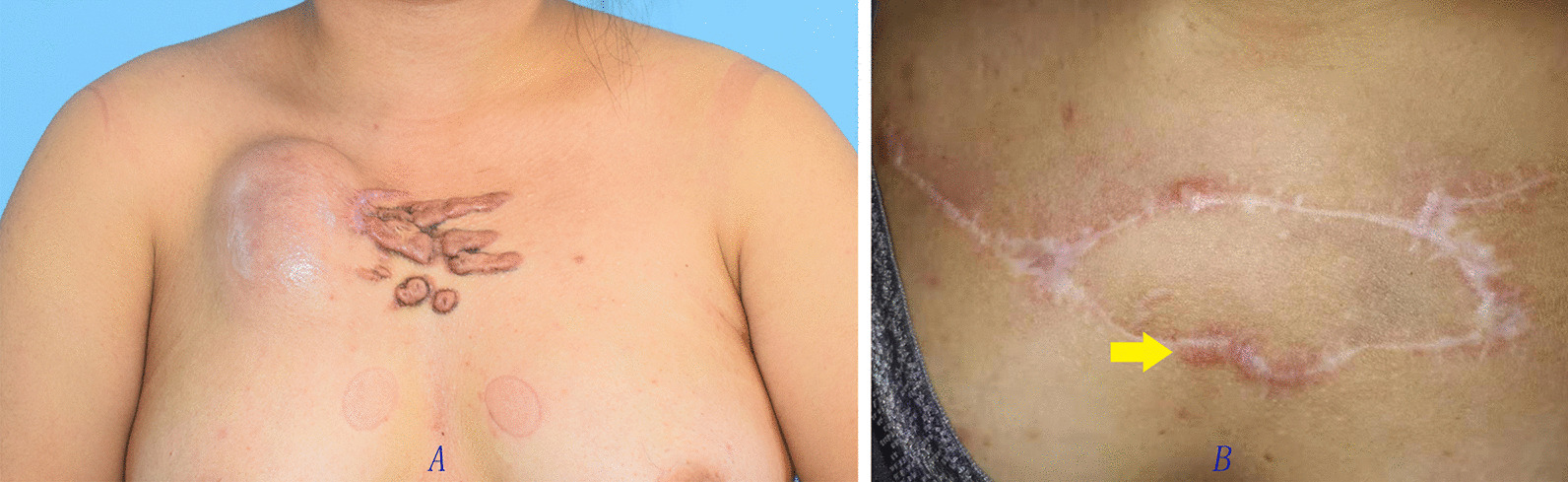
Chest keloid treated with EPIPF

Data of 15 patients were brought into the retrospective analysis. All the surgeries were successful in 15 cases. Only one postoperative complication was reported which infection under the scab occurred after second surgery, it was cured by wound care. The sizes of the EPIPF were from 5 cm × 9 cm to 8 cm × 14 cm. No necrosis of expanded skin flap occurred. One patient had slight skin dimpling when touching by hand. Four patients had small bulge. Of the 15 patients, 11 were cured with slight ordinary scar (Figs. 1, 2, 3, 4) and four were partially cured with a portion of scar hypertrophy. (Fig. 5) No recurrence case was reported until we wrote this report. Esthetic satisfaction results showed that 11 patients thought that the esthetic result was good (73.7%), and 4 patients thought the result was acceptable (26.7%). None patient was dissatisfied.

## Discussion

Keloids are often caused by skin injuries like trauma or surgery, chronic tissue infection or even no reason. The formation of keloids is affected by local skin tension, family history, age, site and even immune function of human body [4]. In all the growing sites of keloid, chest is the most common site because of its possession of skin tension [8]. In addition, the skin tissue in chest has much less mobility than other sites of human body.

In recent years, the most common treatment for chest keloids is resection followed by radiotherapy [9]. To resect the keloids completely, clinicians needed to use skin graft, local flaps or perforators for the reconstruction of wound after resection. The reported surgical methods for very big keloid include pre-cut and pre-radiotherapy with skin graft [6], intercostal perforator skin flap, [7] microsurgical SCIP skin flap transplantation [10]. However, sometimes the size of keloids can be so big that it is hard for surgeons cover the wound only by using local skin flap or perforator skin flap even with microsurgery. At that time, clinicians could choose surgery strategies like skin grafting or incomplete resection in time of without enough tissue nearby to reconstruct the wound. But these surgery methods have some apparently disadvantages. For example, skin graft can be much thinner than flaps, which means it can be much easier to be affected by radiotherapy [6]. Incomplete resection means more chance of recurrence because of keloid tissue presence. Microsurgery with bigger SCIP skin flap may also be the choice, but for SCIP skin flap transplantation, the surgical process is more complicated and skin flap failure may be encountered in some patients which is an unfortunate disaster for the patient. On the other hand, longer scar in the low abdomen can seldom be accepted by the patient.

Although the usage of pre-expanded parasternal intercostal perforator in treating enormous chest keloids are divided into two surgery phases, which makes it seems a bit complicated than complete resection with skin graft or local flap, but this surgery strategy has much more advantages, especially when we are dealing with large keloids or very big female chest keloid. Firstly, expanded perforator flap means its size can be much bigger than a normal perforator flap, which can cover a bigger wound after the resection [11]. Secondly, because donor sites of pre-expanded perforators are always come from locations besides the keloids, the thickness and appearance can be much similar between the donor and receptor site [11]. In addition, compared to the thickness of skin grafts, pre-expanded perforators are much resistant to radiotherapy, which means less possibility of necrosis. Local skin flap or perforate skin flap transplantation in chest is more difficult in female because of presence of breasts. Breast deformation secondary to nearby skin donating often occurs in clinical practice. Such complication can be avoided with EPIPF.

In the surgery aspect, tissue expander implantation (Phase 1 surgery) is a surgery with almost no difficulty and it is a process that cause quite small trauma to patients, which makes it a small surgery for both patients and surgeons. In the normal surgery of keloid resection covered with normal perforators, the biggest difficulty for surgeons is to separate the perforator from deeper tissue without hurting the perforator vessels. However, because the tissue expander often be implanted for 3–4 months, perforator vessels will be given time to grow larger than it was because of compensatory enlargement for supplying more expanded skin tissue. Also the deeper side of pre-expanded perforators can be enveloped by capsule, which is quite easy to identify and separate the perforators without hurting the vessels. On the other hand, expansion process is also a delaying process for the flap and the blood supply of the flap is augmented [12]. That is the reason why no surgical failure of this surgery strategy is found in these reported cases.

Tissue expansion has become an ordinary surgical method in clinical practice. It also helps to decrease the difficulties of parasternal intercostal perforator flap transplantation. But the process is still complicated and some principles should be followed in order to achieve the best surgical results. (1) Considerable preoperative design. The size and the shape of the keloid are not the same in different patients. The shapes of the keloid are often irregular. But the routes of the parasternal intercostal perforator vessels are definite. The surgeon should plan carefully about the size of the expander, the size of the expanded skin flap, the blood supply of the flap and its rotation site. Normal skin around the keloid can be moved to cover some area of the wound after keloid removing, the needed skin flap size may be smaller than keloid size. Besides, with very large irregular keloid, many expanders will be needed. The surgeon should determine which expander is for EPIPF and which is used for normal expanded skin flap at that time. (2) Sufficient expansion. To generate enough skin tissue to cover the wound after keloid resection, the implanted expander should be sufficiently expanded. Over expansion is sometimes needed. (3) Skin with some small keloids can be expanded. If there is not enough healthy skin tissue beside the keloid mass, skin with some small keloids can also be used as expansion site. Long time expansion pressure can cause these small keloids atrophy and can then be cured by radiotherapy. (4) Change irregular wound into regular wound. The keloids are often irregular in the chest and the wounds after keloid resection are also irregular. By changing the irregular shape of the wound into regular wound, the expanded skin flap can be easily designed. (5) To ensure that the parasternal intercostal perforator vessels are covered by appropriate amount tissue during skin flap transplantation. The blood supply of expanded skin is unstable because of vessel redistribution, stable blood supply and venous drainage is important in guarantee skin flap survival. During skin flap dissection, appropriate amount of tissue around the vessel pedicle should be left to ensure minimal disturbance of the parasternal intercostal perforator vessels.

## Conclusion

Pre-expanded parasternal intercostal perforators skin flap is an effective and safe surgery method for reconstruction of wide range of wound as a result of keloid resection. It offers another useful surgical choice for large keloid treatment.

## Data Availability

All data used by or generated in this study is available from the corresponding author upon reasonable request.

## Abbreviations

EPIPF: Expanded parasternal intercostal perforator flap
SCIP: Superficial circumflex iliac artery perforator

## References

[1] Marneros AG, Krieg T (2004). Keloids: clinical diagnosis, pathogenesis, and therapy options. J Dtsch Dermatol Ges.

[2] Lee SS, Yosipovitch G, Chan YH (2004). Pruritus, pain and small nerve fiber function in keloids: a controlled study. J Am Acad Dermatol.

[3] Berman B, Maderal A, Raphael B (2017). Keloids and hypertrophic scars: pathophysiology, classification, and treatment. Dermatol Surg.

[4] Wolfram D, Tzankov A, Pülzl P (2009). Hypertrophic scars and keloids—a review of their pathophysiology, risk factors, and therapeutic management. Dermatol Surg.

[5] Froelich K, Staudenmaier R, Kleinsasser N, Hagen R (2007). Therapy of auricular keloids: review of different treatment modalities and proposal for a therapeutic algorithm. Eur Arch Otorhinolaryngol.

[6] Wenbo LI, Youbin WANG, Xiaojun WANG, Zhifei LIU (2014). A keloid edge precut, preradiotherapy method in large keloid skin graft treatment. Dermatol Surg.

[7] Song KX, Wang YB, Zhang MZ, Wang XJ (2018). A parasternal intercostal perforator flap for esthetic reconstruction after complete chest keloid resection: a retrospective observational cohort study. J Cosmet Dermatol.

[8] Park TH, Seo SW, Kim JK, Chang CH (2011). Management of chest keloids. J Cardio-thorac Surg.

[9] Xiao L, Mingzi Z, Yang W, Ru Z, Youbin W, Xiaojun W. Algorithm of chest wall keloid treatment. Medicine. 2016; 95:35. 10.1097/MD.0000000000004684 PMC5008580 27583896

[10] Zeng A, Song K, Zhang M, Men Q, Wang Y, Zhu L, Liu Z (2017). The "sandwich therapy": a microsurgical integrated approach for presternal keloid treatment. Ann Plast Surg.

[11] Albarah A, Kishk T, Megahed M, Elsakka D, Ghareeb F (2010). Pre-expanded extended island parascapular flap for reconstruction of post-burn neck contracture. Ann Burns Fire Disasters.

[12] Cherry GW, Austed ED, Pasyk KA (1983). Increased survival and vascularity of random pattern skin flaps elevated in controlled expanded skin. Plast Reconstr Surg.

